# Type of Family Support for Infant and Toddler Care That Relieves Parenting Stress: Does the Number of Children Matter?

**DOI:** 10.3390/healthcare11030421

**Published:** 2023-02-01

**Authors:** Xiumin Hong, Wenting Zhu, Sijie Zhao

**Affiliations:** 1 Faculty of Education, Beijing Normal University, Beijing 100875, China; 2 School of Government, Beijing Normal University, Beijing 100875, China

**Keywords:** infant and toddler care, family support, parenting stress, children

## Abstract

The present study aimed to investigate the status and relationships between family support for infant and toddler care and parenting stress, and to explore differences related to the number of children in the families. We conducted a survey among 13,390 Chinese parents who were randomly sampled from six provinces of China. Descriptive analysis, multivariate analysis of variance, and regression analysis indicated that (1) current family support for infant and toddler care in China is insufficient; (2) most Chinese parents reported moderate parenting stress, with the highest scores given for parental distress, followed by difficult child, and parent–child dysfunctional interaction; (3) the larger the number of children in the family, the less the family support for infant and toddler care, the greater the parenting stress; (4) there was a difference between the effects of family support for infant and toddler care on relieving parenting stress among families with different numbers of children. These findings indicate that different types of family support for infant and toddler care should be provided for families with different numbers of children, to ease parenting stress and promote the implementation of the government’s current fertility policy.

## 1. Introduction

Supporting the well-being of children is a priority of the national welfare policy [1]. The establishment and improvement of a child care system for children’s well-being is an especially important sign of the country’s socioeconomic development. With continuous changes in population and family structure, a “care deficit” for infants and toddlers has become a global social problem [2]. More and more mothers of young children enter the labor force, and families face challenges regarding the balance between parenting and work [3,4]. Traditional systems of infant and toddler care have been weakened, and members of the “sandwich generation” who need to take care of old people and young children shoulder an increased burden of responsibility for care [5,6,7]. The care of young children under 3 years old in China has traditionally been undertaken within the family; external sources of family support for infant and toddler care are particularly scarce [8,9]. Since the enactment of the two-child policy and the newly enacted three-child policy in mainland China [7,10], the traditional mother-dominated childcare model cannot be sustained, and the problem of caring for children under 3 years old needs to be solved urgently.

In the context of limited family support for infant and toddler care, parents inevitably encounter practical problems such as work–family conflict in the process of parenting [11,12,13,14]. Parenting stress can occur when changes and challenges relating to these problems surpass parents’ competency and available resources [15], which can not only affect family well-being and child development [16,17]. In the long term, this pattern may also affect China’s fertility levels [18,19].

Previous studies have investigated family support for childcare and parenting stress separately [7,20]. However, there is a lack of research in the context of the three-child policy [12,21], and previous studies have not considered the characteristics of family structure changes [22]. In addition, there is a lack of comprehensive analysis of infant and toddler care support from the perspective of the family, and the impact on parenting stress is also unclear. On this basis, the current study sought to explore the perceptions of family support for infant and toddler care and parenting stress, and which types of family support for infant and toddler care have an impact on the parenting stress of families with different numbers of children. This study aimed to provide empirical evidence that could be useful for improving infant and toddler care support policies, ensuring the well-beingof women and children, and enriching relevant knowledge in the field of child care.

## 2. Literature Review

### 2.1. Types of Family Support for Infant and Toddler Care in China

The efficient strategic provision of family support for infant and toddler care is critical for promoting family well-being [23]. As increasing attention is paid to infant and toddler care internationally, China has been actively developing more comprehensive infant and toddler care support systems [24,25]. In April 2019, the General Office of the State Council of the People’s Republic of China issued Guiding Opinions on Promoting the Development of Care Services for Infants and Toddlers under the Age of 3 (referred to as “Guiding Opinions”) [26], which involved a clear proposal to strengthen the support provided to families for infant and toddler care. These supports include newborn visits, parental leave, childcare services, parent training, and other relevant policy measures to support family and child development in a comprehensive way.

With the strengthening of policy guidance on infant and toddler care, research attention regarding this field is increasing. Some studies have revealed a mismatch between the demand and supply of infant and toddler care support [22,27]. However, previous studies have lacked a systematic theoretical framework for integrating types of infant and toddler care support comprehensively. In the context of the current three-child policy in China, the investigation of infant and toddler care should start from the perspective of direct stakeholders, and examine the “voice” of families in infant and toddler care through the direct perceptions and self-reported needs of families. It is important to assess which new policies and reforms regarding infant and toddler care support in China are effective and “demand-led” [28].

Existence, relatedness, and growth (ERG) theory assumes that human beings have three core needs that each individual strives to fulfill, from low- to high-level requirements. On the basis of ERG theory, family’s needs could be divided into three categories, including obtaining the means of material existence, maintaining interpersonal relatedness to significant others, and seeking opportunities for a unique personal development and growth [29,30,31]. The satisfaction of low-level needs often stimulates a strong desire for high-level needs, leading to more energy being used to pursue high-level needs [32,33]. ERG theory divides people’s needs into existence, relatedness, and growth, which is helpful for informing comprehensive empirical investigations of infant and toddler care from the perspective of the family.

In the current study, family support for infant and toddler care were considered to be an important part of life-cycle service management [26]. Referring to ERG theory, family support for infant and toddler care could be divided into various types. First, existence needs include the satisfaction of various forms of material and physiological desires for safety. The needs of family support for infant and toddler care at the level of existence needs include newborn visits, growth inspection, and disease prevention to ensure the provision of the basic requirements of life for young children and the healthy development of mothers [34,35]. Second, relatedness needs refer to the emotional satisfaction obtained from social respect and recognition, and the need for help and support when faced with difficulties. Previous studies reported that parental leave, childcare allowances, and flexible working hours are conducive to meeting the needs of parents in this regard [36,37]. Third, growth needs refer to requirements for individual improvement and realization. In the domain of family support for infant and toddler care, growth needs not only refer to needs for various forms of childcare services, but also include the needs of scientific parenting support services for infants and toddlers [38]. ERG theory suggests that the needs of family support for infant and toddler care would be likely to exhibit diverse characteristics. In addition, under the three-child policy, families with different numbers of children may have different demands for care support [38]. These potential differences warrant further investigation.

### 2.2. Parenting Stress in the Context of China’s Three-Child Policy

Raising children involves both joyful moments and challenges over many years [39]. When the burdens outweigh the rewards of parenting, mothers and fathers may find it difficult to carry on within this key social role, and may experience parenting stress [40]. Common stressors in parenting include overload, parent–child relationship strain, caregiving strain or role captivity, financial strain, and work–family conflict [15,41]. Modern parents may face new types of challenges that previous generations did not experience. Previous studies have reported that, in the context of the three-child policy, the increase in the number of children may aggravate the parenting stress experienced by families [12,21], and consequently have negative impacts on child development, family motivation and parenting satisfaction [42,43]. Therefore, it is important to elucidate the current situation regarding parenting stress during the implementation of the three-child policy.

Reliable and valid instruments are necessary for measuring parenting stress effectively. Abidin (1990) [44] developed the Parental Stress Index (PSI) to measure parenting stress in terms of three factors: parenting distress, parent–child dysfunctional interaction, and difficult child. Studies in China and other countries have reported on both the reliability and validity of the PSI [45,46,47]. From this perspective, the PSI provides a measure of parents’ stress levels associated with the demands of parenting, and an approach for identifying dysfunctional parenting.

From a family systems perspective [48], the addition of a family member requires reorganization of the family system, indicating that bringing the first child and subsequent children into the family requires adjustments for the parents. Consequently, parents with one or more children are likely to experience parenting stress. Krieg (2007) [49] reported that mothers with one or two children experienced equivalent levels of parenting stress, which increased among all mothers from prenatal to postnatal assessment. However, other studies reported that mothers’ parenting stress in families with more children often involved greater demands when caring for multiple children at the same time [12,50,51], and raising multiple children is reported to be associated with greater adjustment difficulties and higher levels of role strain [52]. Thus, the arrival of children may affect the family’s level of parenting stress. However, with the increase in the number of children in the context of China’s three-child policy, there is currently a need to clarify whether levels of parenting stress are increasing.

### 2.3. Relationships between Types of Family Support for Infant and Toddler Care and Parenting Stress in Families with Different Numbers of Children

According to the buffering model [53], social support plays a role in the physical and mental health of individuals through the elimination of stress. Social support can buffer the negative impact of stressful events on the physical and mental conditions of individuals and maintain or improve individuals’ physical and mental health. For this reason, providing care support that meets the needs of families is conducive to easing the parenting stress of families with infants and toddlers [51]. The theory of work–life balance demonstrates that work–life balance plays an important role in individual well-being such as health satisfaction, family satisfaction, and overall life satisfaction [54]. Family support for infant and toddler care is considered to be an important tool to help families balance work and life, which can relieve the stress of parenting [21,41]. Previous studies have examined the impact of certain types of family support for infant and toddler care on parenting stress. For instance, home-visiting sessions have been suggested to moderate mothers’ anxiety levels at delivery, and parenting stress after 3 months [55]. Parental leave has been shown to contribute to various health outcomes in parents and is conducive to reducing their perceived parenting stress [56,57]. Additionally, the provision of high-quality childcare services is reported to have a wide range of beneficial effects for alleviating work–family conflict and parenting stress, and in promoting parental well-being [58,59,60]. However, because these previous studies did not integrate and compare multiple types of family support for infant and toddler care systematically, it remains unclear how to provide targeted support to families to help them deal with parenting stress most effectively.

Previous studies have suggested that families with multiple children are likely to experience more stress and greater challenges than those with one child and require more care support [7,21]. Families with different numbers of children may have different needs for care support not only in quantity, but also in content, and different kinds of care support may have different effects on relieving parenting stress. First-time parents may lack knowledge and skills regarding children’s physical and psychological development, and may experience greater uncertainty about ensuring basic life care for infants and toddlers [61]. Although parents of two or more children will go through similar stages, each child is an independent individual with unique personality and temperament, so parents must face new setbacks and challenges. At the same time, because raising more children means spending more time, emotional resources, and money, parenting stress in multi-child families may be more strongly influenced by relatedness support and growth support [51,62]. Overall, this previous research suggests the importance of considering the number of children when examining the relationships between family support for infant and toddler care and parenting stress in the current study.

### 2.4. The Context of This Study

With the rapid development of China’s economic and social policies and labor market, dramatic changes have taken place in the family. These changes have led to the weakening of traditional family-based care systems for children under the age of 3, increasing the demand for alternative social childcare [63]. In the context of China’s three-child policy, as the number of children increases, the challenges and pressures of parenting are likely to be exacerbated. A relatively complete family support for infant and toddler care would be expected to ease the worries and pressures of family parenting. Therefore, the current study aimed not only to understand the current situation of family support for infant and toddler care and parenting stress, but also to discuss the relationship between the two. To solve the problem described above, we conducted a national survey to explore the following questions:

1. What types of support for infant and toddler care do Chinese families perceive exist? Are there differences among families with different numbers of children?
2. What are the characteristics of parenting stress experienced by Chinese families? Are there differences among families with different numbers of children?
3. Which types of family support for infant and toddler care could substantially alleviate parenting stress among families with different numbers of children?

## 3. Materials and Methods

### 3.1. Participants

A stratified cluster sampling approach was employed to recruit families with children under 3 years old. This study was part of a project commissioned by the National Health Commission of the People’s Republic of China, and the survey was conducted in representative provinces across the country. First, we randomly selected six provinces: Beijing, Shanghai, Shanxi, Henan, Guizhou, and Yunnan. These provinces represent the Eastern, Central, and Western regions of China, and reflect China’s different economic levels and educational development levels. Second, we randomly sampled two cities from each participating province, resulting in 12 participating cities. Third, for the above-mentioned target cities, with the assistance of the local health department, e-questionnaires were distributed via community WeChat groups, and parents with children under the age of 3 were invited to participate in the survey. Finally, a total of 13,390 valid questionnaires were returned for this national survey. The sample covered the Eastern (34.4%), Central (39.4%), and Western (26.2%) regions, providing a geographically representative Chinese population. Among the respondents, 33.1% were located in rural areas and 66.9% of families were located in urban. The sample included 5503 (41.1%) one-child families, 6454 (48.2%) two-children families, and 1443 (10.7%) three or more-children families.

### 3.2. Measurement

#### 3.2.1. Family Support for Infant and Toddler Care Questionnaire

The family support for infant and toddler care questionnaire was designed as a nationally representative survey to investigate infant and toddler care support from families’ perspective in China. This questionnaire includes two sections: demographic information, and family support for infant and toddler care. 

The first section of the questionnaire mainly investigated the demographic information of families with children under 3 years old. In addition to the geographic region (Eastern = 1, Central = 2, and Western = 3), living area (rural area = 0, urban area = 1), and number of children (one child = 1, two children = 2, three or more children = 3) presented above, we also investigated the following information and they were coded as categorical data. Monthly family income was measured on a three-point scale: 1 = under CNY 5000, 2 = CNY 5001–CNY 10,000, 3 = CNY 10,001 or above. Education attainment was measured on a 3-point scale: 1 = senior high school education or below, 2 = up to 3 years of college education, and 3 = 4 years or more of university study. According to Lu (2002), occupational level in China can be categorized into nine groups. Parental occupation ratings were assigned a code number from 1 to 9, with larger numbers indicating greater occupational prestige. In addition, family work status was also examined (1 = both out of work, 2 = only one working, 3 = dual earners). Family income, maternal and paternal education, and paternal and maternal occupations were standardized (Z-score), and SES was then identified as an observed construct using five standard scores. This section of the survey also asked about the child’s primary caregiver (0= non-parental care, 1 = parental care).

In the second section, investigation of family support for infant and toddler care was mainly based on the ERG theory and the Guiding Opinions [26]. ERG theory proposes that human needs are composed of existence, relatedness, and growth needs. On this basis, in the current study we proposed a three-dimensional survey framework for family support for infant and toddler care. First, existence support consisted of three items, including child health-related support, to ensure the basic needs of infants and toddlers for basic life care and healthy development (e.g., “newborn visits”); second, relatedness support consists of three items (e.g., “parental leave”), mainly by providing support to help families better balance family and work, and being respected and recognized by society; third, growth support consists of three items (e.g., “childcare services”), including childcare services for infants and toddlers and scientific parenting guidance services, to meet the needs of families with young children at the level of development needs. This is the dichotomous (yes/no) scale. In this study, Cronbach’s alpha for the subscales of the infant and toddler care support questionnaire ranged from 0.54 to 0.60.

#### 3.2.2. Parenting Stress

Parenting stress was assessed using the Parenting Stress Index-Short Form (PSI-SF) [64]. The PSI-SF has previously been validated in Chinese samples [62] and has demonstrated good psychometric properties. The PSI-SF consists of 36 items and three dimensions: parental distress (e.g., “I often have the feeling that I cannot handle things very well”, 12 items), difficult child (e.g., “My child seems to cry or fuss more often than most children”, 12 items), and parent–child dysfunctional interaction (e.g., “Sometimes I feel my child doesn’t like me and doesn’t want to be close to me”, 12 items). Parents responded to the items on a five-point Likert scale ranging from (1) strongly disagree to (5) strongly agree. In this study, Cronbach’s alpha for the three subscales of the PSI-SF ranged from 0.93 to 0.94.

### 3.3. Procedures

Ethical procedures were followed throughout the research process. First, we used e-questionnaire for investigation, which proved to have good reliability [65]. In order to ensure that the e-questionnaire can be understood by the participants, we invited three experts in the field of early childhood education and five parents of infants and toddlers to conduct a pre-test before the formal use of the electronic questionnaire to avoid the existence of language wording difficult to understand or ambiguous expressions. Second, informed consent was obtained from participants after they received information about the research objectives and an assurance that the information collected would be used solely for research purposes. After consent was obtained, a link to an online questionnaire platform (“Wenjuanxing”) was sent to all participants. The e-questionnaire required complete responses for submission. Thus, if parents did not complete all of the questions, their responses could not be successfully submitted and the online system automatically prompted them to complete the whole questionnaire. A total of 13,552 e-questionnaires were returned. We excluded 162 questionnaires for the following reasons: (1) reporting information about children over 3 years old, or (2) over 90% of the responses to scaled questions were identical. In total, 13,390 valid questionnaires were included in the final dataset (98.9% effective rate).

### 3.4. Data Analysis

The survey data were collected and analyzed using IBM SPSS 25.0. For the first and second research question, we provided descriptive statistics to reveal the types of infant and toddler care support and parenting stress perceived by Chinese families, and used multivariate analysis of variance (MANOVA) to analyze the difference by families with different numbers of children. To answer question three, correlation analyses between demographic variables (geographic region, living area, family SES, family work status, and primary caregiver) and dependent variables were first conducted, so that the relevant variables could be controlled in further analyses. We then explored which types of infant and toddler care support were perceived as playing a significant role in relieving parenting stress in families with different numbers of children using regression analyses.

## 4. Results

### 4.1. Family Support for Infant and Toddler Care in China

#### 4.1.1. Current Status of Family Support for Infant and Toddler Care in China

The perceived level of currently available family support for infant and toddler care was low. Regarding the status of family support for infant and toddler care, the score for relatedness support was highest (mean [*M*] = 0.45, standard deviation [*SD*] = 0.30), followed by existence support (*M* = 0.36, *SD* = 0.30), and the lowest was the growth support (*M* = 0.33, *SD* = 0.31).

**Figure 1:**
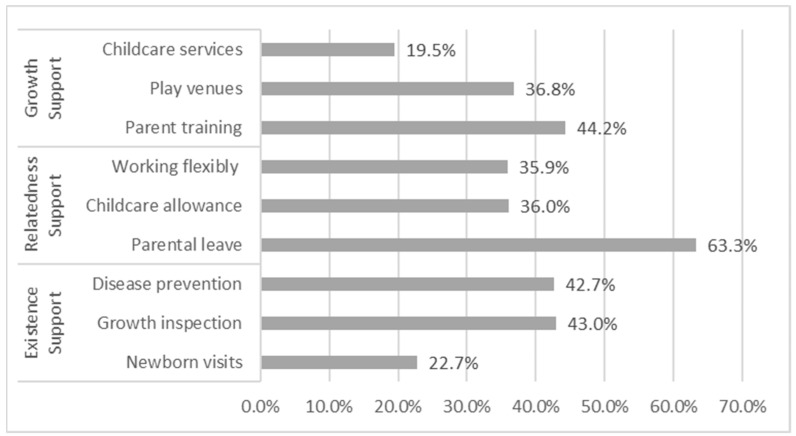
Family support for infant and toddler care.

Specifically, regarding existence support, approximately 22.7% of Chinese families received newborn visits, 43.0% of families received child growth inspection, 42.7% of families received child disease prevention support (see Figure 1). In terms of relatedness support, 63.3% of families received parental leave, 36.0% of the parents received childcare allowance, and 35.9% of the parents had flexible working hours during the maternity period. Regarding growth support, parent training was the most commonly reported (44.2%), followed by play venues (36.8%), and last was childcare services (19.5%).

#### 4.1.2. Family Support for Infant and Toddler Care among Families with Different Numbers of Children

MANOVAs were conducted to explore the possible effects of the number of children (one child, two children, and three or more children) on the perceived infant and toddler care support. The results revealed a significant major effect of the number of children, Wilks’ λ = 0.985, *F*(6, 26,770) = 32.71, *p* < 0.001, η^2^ = 0.01 (i.e., small effect size, η^2^ = 0.01; moderate effect size, η^2^ = 0.06, and large effect size, η^2^ = 0.14). Additionally, differences in the number of children were only found in relatedness support, *F*(2, 13,387) = 80.29, *p* < 0.001, η^2^ = 0.01. However, there was no significant effect for existence support, *F*(2, 13,387) = 14.43, *p* < 0.001, η^2^ = 0.002, and growth support, *F*(2, 13,387) = 4.97, *p* < 0.01, η^2^ = 0.001. Finally, the post-hoc analysis indicated that the perceived support differed by the number of children (See Table 1). The support among three-child families was lower than that among two-child and one-child families, and the support among two-child families was lower than that among one-child families. The results revealed that the greater the number of children in the family, the less support the families perceived they had.

### 4.2. Parenting Stress in Chinese Families with Infants and Toddlers

#### 4.2.1. Descriptive Analysis of Parenting Stress in Chinese Families

Most Chinese parents reported experiencing moderate stress, with the means of the overall score being slightly higher than 3.0. Parental distress (*M* = 3.50, *SD* = 0.78) had the highest score, followed by difficult child (*M* = 3.18, *SD* = 0.71), and parent–child dysfunctional interaction (*M* = 2.86, *SD* = 0.84). Thus, the results indicated that among Chinese families, caregivers most commonly experienced stress related to the challenges of parenting, whereas relatively few caregivers experienced stress related to parent–child dysfunctional interaction.

#### 4.2.2. Parenting Stress among Families with Different Numbers of Children

We conducted MANOVAs to explore the possible effects of the number of children on parenting stress. The results revealed a significant effect of the number of children (one child, two children, and three or more children). Specifically, we examined main effects of overall satisfaction and found a significant effect of the number of children, Wilks’ λ = 0.962, *F*(6, 26,770) = 40.29, *p* < 0.001, η^2^ = 0.01, and the power to detect the effect was 1. Next, differences related to the number of children were found for two constructs: difficult child, *F*(2, 13,387) = 112.98, *p* < 0.001, η^2^ = 0.02 and parent–child dysfunctional interaction, *F*(2, 13,387) = 96.90, *p* < 0.001, η^2^ = 0.01. However, there was no significant effect on parental distress, *F*(2, 13,387) = 31.59, *p* < 0.001, η^2^ = 0.005. Finally, a post-hoc analysis indicated that parenting stress was differentiated by the number of children (See Table 2). The pressure experienced by parents in three-child families was found to be significantly greater than that experienced by parents in two- and one-child families (*p*s < 0.001), and the pressure in two-child families was significantly greater than that in one-child families (*p*s < 0.001). Overall, the results revealed that a greater number of children in the family was associated with higher parenting stress.

### 4.3. Effect of Family Support for Infant and Toddler Care on Parenting Stress: Difference in the Number of Children

First, Pearson’s correlation analysis was conducted to explore the correlation between control variables, family support for infant and toddler care, and parenting stress in China. As shown in Table 3, demographic variables of geographic region, living area, family SES, family work status, and primary caregiver were significantly related to parenting stress and controlled for in further analyses (*p*s < 0.01). Meanwhile, eight of the family support for infant and toddler care were significantly associated with parenting stress and were also included in subsequent regression models. In addition, we verified the common method bias using the Harman single-factor test.

Next, linear regression with the enter method was then conducted to examine the impact of family support for infant and toddler care on parenting stress. The independent variables were nested at two levels: (1) demographic variables: geographic region (Eastern = 1, Central = 2, and Western = 3), living area (rural area = 0, urban area = 1), family SES (low income = 1, medium income = 2, high income = 3), family work status (1 = both out of work, 2 = only one working, 3 = dual earners), and primary caregiver (0 = non-parental care, 1 = parental care); (2) family support for infant and toddler care: existence support (newborn visits, growth inspection, and disease prevention); relatedness support (parental leave, childcare allowance, working flexibly); growth support (play venues, childcare services). Accordingly, demographic variables were entered in Step 1, and eight family supports for infant and toddler care as independent variables were entered in Step 2. To further examine the differences among families with different numbers of children, three separate regression models were constructed. The regression model results are shown in Table 4.

For the one-child families, basic existence support had the most significant effect on relieving parenting stress, including all support for this dimension: newborn visits (*β* = −0.08, *p* < 0.001), growth inspection (*β* = −0.09, *p* < 0.001), and disease prevention (*β* = −0.06, *p* < 0.001). Regarding growth support, play venues (*β* = −0.04, *p* < 0.01) and childcare services (*β* = −0.08, *p* < 0.001) also showed a significant effect.

For the families with two children, existence support also had the most significant effect on relieving parenting stress, newborn visits (*β* = −0.03, *p* < 0.001), growth inspection (*β* = −0.05, *p* < 0.01), and disease prevention (*β* = −0.05, *p* < 0.001). In relatedness support, flexible working hours significantly reduced parenting stress (*β* = −0.04, *p* < 0.01). Regarding growth support, play venues (*β* = −0.06, *p* < 0.001) and childcare services (*β* = −0.07, *p* < 0.001) showed a significant effect.

For families with three or more children, the effect of existence support and relatedness support on parenting stress was no longer significant, while the effect for growth support was still significant. These families reported needing play venues for children (*β* = −0.10, *p* < 0.05). Additionally, the effect of social childcare services was also significant (*β* = −0.12, *p* < 0.01).

In summary, the regression analyses revealed that family supports for infant and toddler care were critical predictors of parenting stress. For families with different numbers of children, growth support has a significant effect on relieving parenting stress. At the same time, there were differences in the relief effects of various types of support on parenting stress. Families with one child tended to report needing more basic existence support, as well as growth support. In addition to existence support and growth support, relatedness support such as flexible working hours was reported to be important for relieving stress in families with two children. Finally, families with three or more children tended to report that they no longer needed basic existence support and relatedness support, but had more of a need for play venues for their children; at the same time, alternative childcare services were reported to be useful for easing parenting pressure.

## 5. Discussion

The current study provided new empirical evidence regarding the current situation and relationships between family support for infant and toddler care and parenting stress in China under the three-child policy. Falling fertility is an increasingly global problem. Despite differences within and between regions, fertility rates are declining rapidly or stabilizing at relatively low levels in most parts of the world. In terms of promoting the implementation of government fertility policies, it is not sufficient to simply encourage families to have children. It is critical to improve support to help families relieve the pressure of child rearing. The current findings extend our understanding of how to improve the support measures of fertility from the perspective of family needs. Additionally, the current findings suggest an approach for improving family support for infant and toddler care in low-fertility societies such as China and many other countries. In the following section, we discuss the current findings and their implications for infant and toddler care and family well-being practice.

### 5.1. Insufficient Family Support for Infant and Toddler Care in Chinese Families

Researchers in the fields of childcare and early education have long been aware that efforts should be made to encourage child-oriented initiatives with a focus on supporting parents [66,67]. However, less attention has been paid to taking steps to ensure that the policies and funding initially put in place to support parental employment (particularly childcare subsidies) also include an integrated and systematic focus on child development. On the basis of ERG theory, a family’s needs could be divided into three categories, including obtaining the means of material existence, maintaining interpersonal relatedness to significant others, and seeking opportunities for unique personal development and growth [29,30,31]. The current study innovatively proposed a three-dimensional framework for infant and toddler care support in Chinese families, and used this framework to investigate the perceived access of infant and toddler care support in Chinese families.

At present, family care is still the main source of care for children under 3 years old in China [68]. Chinese families, especially mothers, bear the main responsibility of childcare, and family support for infant and toddler care is scarce. In addition, the current results revealed a short supply in family support for infant and toddler care. Liu et al. (2022) [69] analyzed access to childcare services for parents under the age of 3 in Sichuan Province, China, and found that there was insufficient adaptability, as well as the coexistence of insufficient supply and demand. Specifically, services were found to be under-utilized; the structure of services provided did not match the needs of families with children under 3 years of age, and the quality of services was low [70]. Under the three-child policy, parents’ demand for family support for infant and toddler care differs by the number of children [38]. However, this study found that families with more children tend to have less family support for infant and toddler care. Inadequate family support for infant and toddler care under the age of three urgently needs to be addressed.

### 5.2. Chinese Families with More Children Experience Greater Parenting Stress

In the current study, we found that Chinese families experienced a moderate level of parenting stress, which is consistent with the survey results in recent years [51,61], and is slightly lower than the earlier survey [71], indicating that with the implementation of the family-friendly policy in China, the parenting stress of Chinese parents has been reduced to a certain extent. Among the types of stress, parental distress was most commonly experienced, followed by difficult child, and parent–child dysfunctional interaction. This pattern is consistent with previous reports [51], indicating that Chinese families face more care and life pressure after becoming parents. Some studies have found that parenting stress occurred as the parents’ feeling of pressure in the process of raising their children [71], or as a negative self-evaluation of parents when the available social support resources cannot meet their parenting needs [72]. Under the three-child policy, Chinese parents need to deal with the weakening of family size and structural diversification, which has weakened the function of raising children and supporting older people, and would also increase the pressure on families to raise children, especially when it comes to parenting responsibilities.

The birth of a new child, and the first weeks, months, and years of a child’s life, are times in which the tension between work obligations and child rearing is particularly acute. According to a survey of 10,155 parents from 21 cities conducted by the China Women’s Federation, over 50.0% of two-child families experienced parenting challenges and stress [73]. For example, 55.1% of parents reported that they did not know how to interact with their two children and 50.0% of parents expressed that they did not know how to deal with sibling relationships. In the current study, a larger number of children was associated with greater parenting stress [12,51]. In general, parenting two or more children involves more stressful challenges than parenting one child because parents often need twice as much time and energy, and are required to deal with new challenges, such as sibling relationships [50,74]. Infants and toddlers require constant attention from adults, whether from a parent or another caregiver, and this constant attention comes at a cost, whether in terms of lost income for the parent or the expense of replacement care. The responsibility of raising babies and toddlers is often exhilarating for parents, but also exhausting [75].

### 5.3. Types of Family Support for Infant and Toddler Care That Relieve Parenting Stress in Families with Different Number of Children

Family support for infant and toddler care is considered to be an important tool to help families balance work and life, which can relieve the stress of parenting [21,41]. The current results revealed that existence support, relatedness support, and growth support have different effects on parenting stress in Chinese families. This requires the separate consideration of families with different numbers of children. For all families with different children, to resolve family and work conflicts and ease the stress of parenting, more non-parental childcare service support may be needed to help families relieve the pressure of childcare [76]. Meanwhile, play venues for their children are also important for relieving stress on parents. Especially for families with three children, these two supports are most necessary. One-child families tended to report needing more basic existence support, as well as growth support for infants and toddlers. This result indicates that new parents lack knowledge and skills about children’s development, and more support is needed to ensure basic care for infants and toddlers [7,21]. For two-child families in the process of parenting their first-born children, parents typically acquire childcare experience, and can make adjustments or improvements in the process of parenting their subsequent children [77]. Although existence support is still important, the arrival of two children can create even greater work–family conflict [78], flexible working hours are also important for families with two children to ease stress.

## 6. Conclusions, Limitations, and Implications

This study aimed to understand the current situation regarding family support for infant and toddler care and parenting stress, and which types of family support for infant and toddler care can relieve parenting stress in the context of China’s three-child policy. The results revealed several key findings. First, the currently available family support for infant and toddler care in China is insufficient. Second, most Chinese parents reported experiencing moderate parenting stress. Among the types of stress, parental distress was most common, followed by difficult child, and parent–child dysfunctional interaction. In addition, a larger number of children in the family was reported to be associated with greater parenting stress but less family support for infant and toddler care. Finally, family support for infant and toddler care could relieve parenting stress in families with different numbers of children.

The current study involved several limitations. First, we examined family support for infant and toddler care as well as parenting stress on the basis of self-reported data from parents. Although we can intuitively understand the experiences of parents, we cannot objectively and comprehensively understand the current situation regarding care support and parental stress. Multidimensional evaluation, instead of sole reliance upon questionnaire data, should be undertaken to avoid response biases such as the social expectation effect from different populations. Future studies should investigate multiple stakeholders and enrich research methods. Second, this study shows that family support for infant and toddler care has a certain effect on parenting stress, but this effect is limited. In addition to family support for infant and toddler care, parenting stress can also be affected by many factors such as parenting style, and socioeconomic status [79,80]. The follow-up studies should be comprehensively considered to provide a basis for policy improvement from the perspective of the integration of childbearing and rearing.

Nevertheless, our findings have many implications for policymakers in China, and internationally. To address the current situation, in which family support for infant and toddler care has been unable to adapt to changing family needs, it is important to adhere to the concept of sustainable development directed by family demand, by promoting supply-side structural reform, accelerating the construction of care support systems for infants and toddlers in China, effectively alleviating the pressure of family rearing and care difficulties, promoting the well-being of families and the healthy growth of children, and helping the implementation of government fertility policies. First, the health and safety of infants and toddlers should be protected. Local governments should provide community maternal and child health services. Through the establishment of comprehensive information systems for community maternal and child health, effective integration and sharing of health information database resources should be undertaken to form a comprehensive and interconnected service information network. Second, parental leave and flexible working policies that can balance family and work should be fully implemented, and husbands and wives should be encouraged to share responsibility for family affairs and childcare. Additionally, family-friendly workplace arrangements should be provided, including flexible working hours and locations, as well as support for women who have had a career break to return to work. Finally, ample play venues and the provision of childcare services should be strengthened to resolve challenges of childcare and parenting. The developing brain exhibits sensitive periods, and while they may vary slightly from child to child, the most sensitive periods are around birth and in the first 3 years. Family policies should be developed that enable children and their parents to thrive, so that all individuals can contribute in a rapidly changing environment.

## Figures and Tables

**Table 1:** Comparison of family support for infant and toddler care with different number of children.

Variable	(I) Number of Children	(J) Number of Children	Mean Difference (I–J)	*SE*
Existence support	One	Two	0.015 **	0.005
		Three or more	0.054 ***	0.010
	Two	One	−0.015 **	0.005
		Three or more	0.039 ***	0.010
	Three or more	One	−0.054 ***	0.010
		Two	−0.039 ***	0.010
Relatedness support	One	Two	0.058 ***	0.005
		Three or more	0.101 ***	0.010
	Two	One	−0.058 ***	0.005
		Three or more	0.043 ***	0.010
	Three or more	One	−0.100 ***	0.010
		Two	−0.043 ***	0.010
Growth support	One	Two	−0.011	0.006
		Three or more	0.019	0.011
	Two	One	0.011	0.006
		Three or more	0.030 *	0.010
	Three or more	One	−0.019	0.010
		Two	−0.030 *	0.010

* *p* < 0.05, ** *p* < 0.01, *** *p* < 0.001.

**Table 2:** Comparison of parenting stress with different number of children.

Variable	(I) Number of Children	(J) Number of Children	Mean Difference (I–J)	*SE*
Parenting distress	One	Two	−0.085 ***	0.014
		Three or more	−0.178 ***	0.027
	Two	One	0.086 ***	0.014
		Three or more	−0.093 ***	0.026
	Three or more	One	0.178 ***	0.027
		Two	0.093 ***	0.026
Difficult child	One	Two	−0.147 ***	0.013
		Three or more	−0.265 ***	0.024
	Two	One	0.147 ***	0.012
		Three or more	−0.118 ***	0.024
	Three or more	One	0.265 ***	0.024
		Two	0.118 ***	0.024
Parent–child dysfunctional interaction	One	Two	−0.193 ***	0.015
		Three or more	−0.328 ***	0.029
	Two	One	0.193 ***	0.015
		Three or more	−0.135 ***	0.028
	Three or more	One	0.328 ***	0.029
		Two	0.135 ***	0.028

*** *p* < 0.001.

**Table 3:** Correlations and descriptive statistics for the main study variables.

Variable	1	2	3	4	5	6	7	8	9	10	11	12	13	14	15
1. Geographic region	–														
2. Living area	−0.03 **	–													
3. Family SES	0.48 **	−0.08 **	–												
4. Work status	0.82 **	−0.07 **	0.56 **	–											
5. Primary caregiver	0.10 **	−0.03 **	0.15 **	0.11 **	–										
6. Newborn visits	0.19 **	−0.05 **	0.16 **	0.20 **	0.06 **	–									
7. Growth inspection	0.18 **	−0.02 *	0.16 **	0.18 *	0.09 **	0.29 **	–								
8. Disease prevention	0.06 **	−0.01	0.05 **	0.05 **	0.07 **	0.16 **	0.26 **	–							
9. Parental leave	0.30 **	−0.03 **	0.28 **	0.44 *	0.05 **	0.15 **	0.18 **	0.07 **	–						
10. Childcare allowance	0.18 **	−0.02 *	0.10 **	0.18 *	0.05 **	0.15 **	0.13 **	0.08 **	0.16 **	–					
11. Working flexibly	0.06 **	−0.02	0.03 **	0.05 **	0.01	0.05 **	0.09 **	0.12 **	0.12 **	0.14 **	–				
12. Parent training	−0.02 *	0.01	−0.03 **	−0.01	−0.03 **	−0.10 **	−0.16 **	−0.20 **	−0.06 **	−0.13 **	−0.14 **	–			
13. Play venues	−0.03 **	0.01	−0.01	−0.05 **	0.03 **	−0.05 **	−0.01	−0.02 *	−0.06 **	−0.05 **	−0.03 **	0.13 **	–		
14. Childcare services	0.07 **	−0.01	0.09 **	0.09 *	0.05 **	0.02 **	−0.05 **	−0.04 **	0.04 *	−0.03 **	−0.05 **	0.12 **	0.13 **	–	
15. Parenting stress	−0.23 **	0.05 **	−0.22 **	−0.24 **	0.14 **	−0.11 **	−0.12 **	−0.07 **	−0.13 **	−0.05 **	−0.03 **	−0.01	−0.05 **	−0.08 **	–

* *p* < 0.05, ** *p* < 0.01.

**Table 4:** Regression results for family support for infant and toddler care predicting parenting stress.

Variable	One-Child Family				Two-Child Family				Three or More Child Family			
	*β*	*SE*	*β*	*SE*	*β*	*SE*	*β*	*SE*	*β*	*SE*	*β*	*SE*
Geographic region	−0.10 ***	0.02	−0.09 ***	0.02	−0.11***	0.02	−0.10 ***	0.02	−0.21 **	0.08	−0.23 **	0.08
Living area	0.04 **	0.01	0.03 **	0.01	0.02	0.01	0.02	0.01	0.13 **	0.03	0.14 **	0.03
Family SES	−0.10 ***	0.01	−0.09 ***	0.01	−0.13 ***	0.01	−0.11 ***	0.01	−0.20 ***	0.03	−0.20 ***	0.03
Work status	−0.08 ***	0.01	−0.05 *	0.01	−0.04 *	0.01	−0.02	0.01	0.10	0.02	0.11	0.03
Primary caregiver	0.12 ***	0.02	0.13 ***	0.02	0.14 ***	0.02	0.15 ***	0.016	0.16 ***	0.10	0.16 ***	0.11
Existence support												
Newborn visits			−0.08 ***	0.03			−0.03 **	0.03			−0.06	0.12
Growth inspection			−0.09 ***	0.03			−0.05 ***	0.02			0.08	0.09
Disease prevention			−0.06 ***	0.02			−0.05 ***	0.02			−0.05	0.09
Relatedness support												
Parental leave			−0.03	0.03			−0.02	0.02			−0.01	0.10
Childcare allowance			−0.01	0.02			0.02	0.02			0.07	0.09
Working flexibly			−0.02	0.02			−0.04 **	0.02			0.02	0.09
Growth support												
Play venues			−0.04 **	0.03			−0.06 ***	0.03			−0.10 *	0.12
Childcare services			−0.08 ***	0.04			−0.07 ***	0.03			−0.12 **	0.16
*R* ^2^	0.08		0.11		0.07		0.09		0.12		0.15	
*ΔR* ^2^			0.03				0.02				0.03	
*F* Value	89.49		45.25		109.16		48.39		10.18		4.97	

* *p* < 0.05, ** *p* < 0.01, *** *p* < 0.001.

## Data Availability

Not applicable.
